# Rectal Metastases from Squamous Cell Carcinoma: A Case Report and Review of the Literature

**DOI:** 10.1155/2012/947524

**Published:** 2012-04-10

**Authors:** S. Cedrés, N. Mulet-Margalef, M. A. Montero, P. Martinez, A. Martínez, E. Felip

**Affiliations:** ^1^Medical Oncology Service, Vall d'Hebron University Hospital, P. Vall d'Hebron 119-129, 08035 Barcelona, Spain; ^2^Pathology Department, Vall d'Hebron University Hospital, 08035 Barcelona, Spain

## Abstract

Non-small-cell lung cancer (NSCLC) represents 85% of lung cancer. The most frequent sites of distant metastasis are the liver, adrenal glands, bones and brain. Gastrointestinal metastases are uncommon and rectal metastases are extremely rare. Here we report a case of squamous cell carcinoma of the lung with rectal metastases.

## 1. Introduction

Non-small-cell lung cancer is a common neoplasm and frequently metastasizes to lung, liver, adrenal, and brain. However, gastrointestinal metastasis development is relatively rare, except esophagus localization by local invasion. Colon metastases have been previously reported but rectal metastases from NSCLC are an extremely rare localization.

## 2. Case

**Figure 1:**
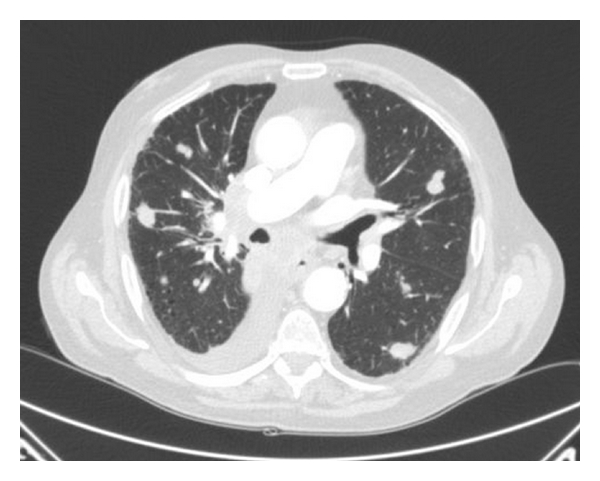
CT scans with multiple bilateral nodules and right pleural effusion.

A 81-year-old former smoker man (45 pack-years) with history of colonic diverticulosis with annual colonoscopy surveillance was presented with haemoptysis and fatigue of several-month duration. As part of the evaluation of these symptoms, a chest X-Ray was performed, which revealed hilar fullness. As result of this finding a computed tomography (CT) was performed and showed a right lower lobe mass with contralateral metastases (Figure 1). The lung biopsy revealed a squamous cell lung carcinoma (SCC) with immunohistochemistry positive for CK5/6 and negative for CDX2, CK7, and TTF1. His final diagnosis was of a T2N2M1 (stage IV) non-small-cell lung cancer (NSCLC).

**Figure 2:**
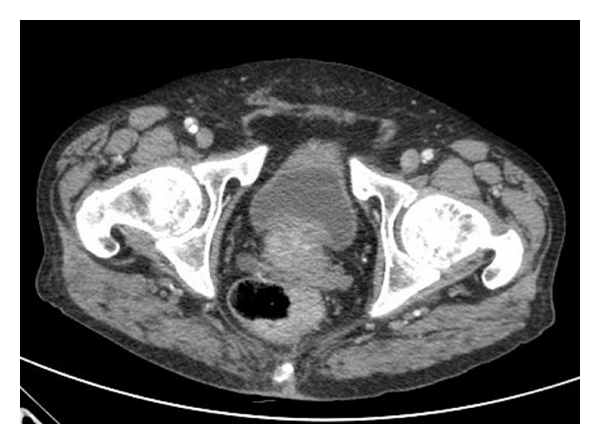
CT scans which show rectal mass.

**Figure 3:**
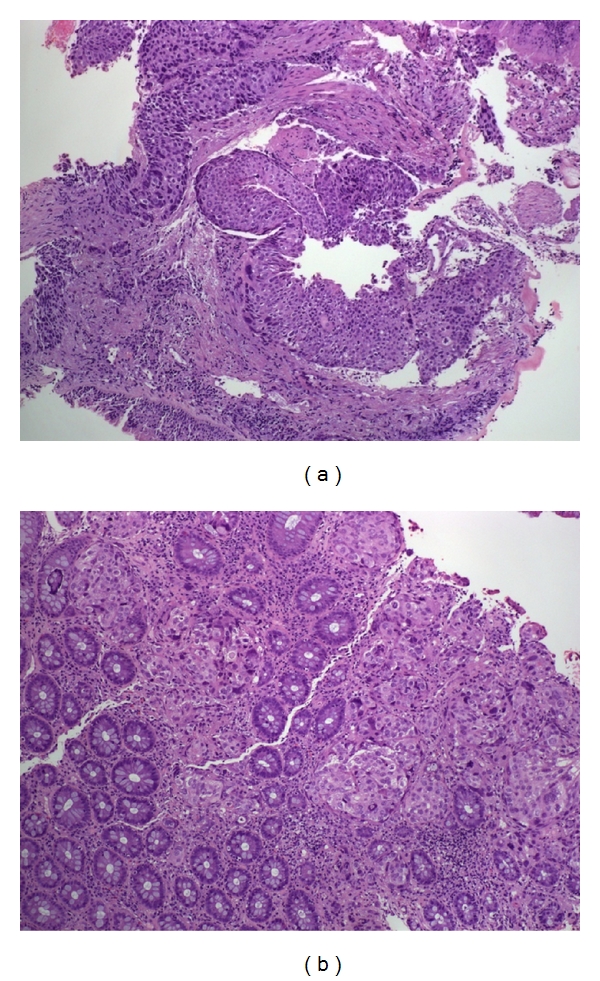
Microscopic findings: (a) (hematoxylin eosin (HE) 10x) primary squamous cell carcinoma. (b) (HE 10x) keratin deposits and digestive glands in rectal metastasis.

Treatment was commenced with chemotherapy (gemcitabine × 4 cycles) achieving stable disease. Three months later the patient presented abdominal pain and the CT scan showed progression of the disease with an increase in size and number of lung nodules and the presence of a thickening of the rectum wall with enlarged regional lymph nodes (Figure 2). Rectum primary tumour was suspected and the study was completed with specific serum tumour markers (CEA and Ca 19.9) with normal value, and a colonoscopy was performed which revealed a 3 cm diameter ulcerated lesion at 8 cm of anal margin. The rectal biopsy was reported as a metastasis from SCC with identical morphological and immunohistochemistry pattern than the lung tumour (CD5/6+, CDX2, TTF1, and CK7−) (Figure 3). Rectal radiotherapy was started for symptomatic control but 5 weeks after the patient died as a consequence of respiratory insufficiency.

## 3. Discussion

Metastatic involvement of the gastrointestinal tract (GI) secondary to NSCLC is relatively frequent in necropsies series (4,3%–18,5%) but rarely recognized clinically [1–4].

Esophagus is the more frequent GI metastatic localization by contiguity invasion and small bowel and stomach are frequent localization as part of haematogenous dissemination. Colonic metastatic from NSCLC has been rarely described and rectal localization is an extremely rare finding [2, 3].

At our knowledge, only two possible cases of rectal metastases from lung cancer have been reported in the literature. In a review of 18 patients with lung cancer initially manifesting as gastrointestinal tract metastases in one centre, there is mention of a possible patient with rectal metastasis [2]. A 73-year female that initially presented rectal bleeding was surgically treated with hemicolectomy. The pathological diagnosis was non-small-cell lung cancer (large cell carcinoma) and four months later patient died due to disease. However, authors did not report about the definitive localization of the bowel metastasis (rectum or left colon). Another report of lung cancer with rectal metastases has been published several years ago. A 50-year-old man was diagnosed with small-cell lung carcinoma and a lobectomy was performed. Two years later, patient presented pain and rectal bleeding and an abdomino perineal resection was performed. The pathology confirmed metastatic small-cell carcinoma and patient died 19 months later of rectal metastasis diagnosis [5]. In a study with autopsy data of 423 patients of primary tumours of the lung, 58 cases (14%) of gastrointestinal metastases were detected, but none of the patientshad rectal involvement [3].

 GI metastases from NSCLC are used to be occasional findings in radiological tests and clinically significant symptoms are rare except for the complications. However, clinical manifestations depend on the site of the metastases with digestive bleeding in case of gastric metastasis, abdominal pain, anemia, and bowel perforation in lower gastrointestinal tract [3, 6].

GI metastasis has been described as synchronous at the initial diagnoses but more frequently metachronous in the context of progression at various sites [1]. The histological type of NSCLC that causes GI metastases varies according to different series, but SCC is responsible of the GI metastasis in around 30% of cases [3].

The lung cancer with intestinal metastasis has been reported to have a poor prognosis with median OS of only 4–8 weeks [3–5]. The treatments modalities depend on the nature of presentation and extent of disease. After resection of colonic metastases anecdotic larger survival was reported [6].

## 4. Conclusion

 GI metastases from NSCLC are more frequent than we observe in clinical daily practice. However, rectal localization is a very uncommon event and, when it happened, worsened the prognosis.

## References

[1] Carroll D, Rajesh PB (2001). Colonic metastases from primary squamous cell carcinoma of the lung. *European Journal of Cardio-Thoracic Surgery*.

[2] Rossi G, Marchioni A, Romagnani E (2007). Primary lung cancer presenting with gastrointestinal tract involvement: clinicopathologic and immunohistochemical features in a series of 18 consecutive cases. *Journal of Thoracic Oncology*.

[3] Antler AS, Ough Y, Pitchumoni CS, Davidian M, Thelmo W (1982). Gastrointestinal metastases from malignant tumors of the lung. *Cancer*.

[4] McNeill PM, Wagman LD, Neifeld JP (1987). Small bowel metastases from primary carcinoma of the lung. *Cancer*.

[5] Johnson AOC, Allen MB (1995). Rectal metastases from small cell lung cancer. *Respiratory Medicine*.

[6] Kim MS, Kook EH, Ahn SH (2009). Gastrointestinal metastasis of lung cancer with special emphasis on a long-term survivor after operation. *Journal of Cancer Research and Clinical Oncology*.

